# Age‐related cholesterol and colorectal cancer progression: Validating squalene epoxidase for high‐risk cases

**DOI:** 10.1111/acel.14152

**Published:** 2024-03-22

**Authors:** Soo Young Jun, Hyang Ran Yoon, Ji‐Yong Yoon, Jeong‐Ju Lee, Ji Yeon Kim, Jin‐Man Kim, Nam‐Soon Kim

**Affiliations:** ^1^ Rare Disease Research Center Korea Research Institute of Bioscience and Biotechnology Daejeon Korea; ^2^ Functional Genomics University of Science and Technology Daejeon Korea; ^3^ Department of Cancer Biology, Cancer Center and Beckman Research Institute City of Hope Duarte California USA; ^4^ Immunotherapy Convergence Research Center Korea Research Institute of Bioscience and Biotechnology Daejeon Korea; ^5^ College of Medicine Chungnam National University Daejeon Korea

**Keywords:** aging, cholesterol, colorectal cancer, machine learning‐based digital image analysis combined with fluorescence‐immunohistochemistry, squalene epoxidase

## Abstract

As people age, the risk and progression of colorectal cancer (CRC), along with cholesterol levels, tend to increase. Nevertheless, epidemiological studies on serum lipids and CRC have produced conflicting results. We previously demonstrated that the reduction of squalene epoxidase (SQLE) due to accumulated cholesterol within cells accelerates CRC progression through the activation of the β‐catenin pathway. This study aimed to investigate the mechanism by which age‐related cholesterol accumulation within tissue accelerates CRC progression and to assess the clinical significance of SQLE in older individuals with elevated CRC risk. Using machine learning‐based digital image analysis with fluorescence‐immunohistochemistry, we assessed SQLE, GSK3β^pS9^ (GSK3β activity inhibition through serine 9 phosphorylation at GSK3β), p53 wild‐type (p53^WT^), and p53 mutant (p53^MT^) levels in CRC tissues. Our analysis revealed a significant reduction in SQLE, p53^WT^, and p53^MT^ and increase in GSK3β^pS9^ levels, all associated with the substantial accumulation of intra‐tissue cholesterol in aged CRCs. Cox analysis underscored the significant influence of SQLE on overall survival and progression‐free survival in grade 2–3 CRC patients aged over 50. SQLE and GSK3β^pS9^ consistently exhibited outstanding prognostic and diagnostic performance, particularly in older individuals. Furthermore, combining SQLE with p53^WT^, p53^MT^, and GSK3β^pS9^ demonstrated a robust diagnostic ability in the older population. In conclusion, we have identified that individuals aged over 50 face an increased risk of CRC progression due to aging‐linked cholesterol accumulation within tissue and the subsequent reduction in SQLE levels. This study also provides valuable biomarkers, including SQLE and GSK3β^pS9^, for older patients at elevated risk of CRC.

## Graphical abstract

As people age, the incidence and mortality of colorectal cancer, as well as high cholesterol levels, increases. Age‐related accumulation of cholesterol accelerates CRC progression in humans by reducing SQLE, inhibiting GSK3β, and suppressing the p53 pathway, thereby providing valuable biomarkers for populations at high risk of CRC.

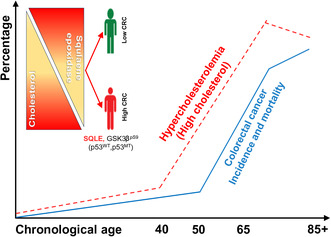

**Abbreviations**
ABCA1: adenosine triphosphate (ATP)‐binding cassette transporter 1
AUC: area under the ROC curve
CRC: colorectal cancer
GSK3βpS9: phosphorylation at serine 9 of GSK3β
LDLR: low‐density lipoprotein receptor
mlDIA‐fmIHC: machine learning‐based digital image with fluorescence‐multiplex immunohistochemistry
OS: overall survival
p53MT: p53 mutant
p53WT: p53 wild‐type
PFS: progression‐free survival
SQLE: squalene epoxidase
timeROC: time‐dependent ROC

## 1 INTRODUCTION

Colorectal cancer (CRC) ranks as the second leading cause of cancer‐related deaths worldwide (WHO, 2020). Moreover, most CRC‐related deaths result from metastases (Li et al., 2020; Wang et al., 2020); therefore, it is imperative to identify biomarkers associated with high‐risk populations vulnerable to malignant CRC, facilitating tailored surveillance and treatment.

CRC incidence and mortality increase with age, not only showing significant growth beyond age 50 (American Cancer Society, 2020); the most prevalent group at risk of CRC is diagnosed between ages 65 and 74 years. Several studies have reported a relationship between a cholesterol/low‐density lipoprotein‐related lifestyle and increased CRC fatality (Di et al., 2023; Farvid et al., 2021; Murphy et al., 2019). In addition, elevated serum cholesterol levels contribute to malignant colonic transformation by inducing tissue hypoxia (Herbey et al., 2005; Paraf et al., 1999). Cholesterol level has also been shown to increase with age (Downer et al., 2014; Hosseini et al., 2019). However, the age‐dependent connection between increased cholesterol and accelerated CRC progression has yet to be established.

Squalene epoxidase (SQLE), a second rate‐limiting enzyme in cholesterol biosynthesis (Chua et al., 2020; Gill et al., 2011), has been reported as a bona fide oncogene in several cancers, including breast (Brown et al., 2016) and liver (Liu et al., 2018). In addition, Gill et al. (2011) and Chua et al. (2020) reported that cholesterol exquisitely regulates the SQLE gene and protein by suppressing SQLE expression and inducing proteasomal degradation of the SQLE protein via MARCHF6‐ubiquitination at the N‐terminal 100 amino acid. Notably, we demonstrated this disconnection under certain pathologic conditions on the expression of the SQLE gene and protein (Jun et al., 2021); SQLE degradation caused by accumulated cholesterol over a certain threshold accelerates CRC progression and metastasis via activating the β‐catenin oncogenic pathway by inhibiting the p53 anti‐tumor suppressor path and GSK3β activity. Our findings of the disruption of the p53/p21 pathway through SQLE reduction are substantiated by the following observations; Overexpression of p53^R273H^ and p53^WT^ hindered the survival of CRC cells (HCT116 and HT29) treated with small interfering RNAs targeting SQLE (siSQLE) under conditions of anoikis resistance. Conversely, the mutant p53 variant pLNCX‐Flag‐p53‐R273H‐mTAD, rendering its transactivation region nonfunctional, did not increase the survival of CRC cells treated with cholesterol or siSQLE under anoikis resistance (Jun et al., 2021).

In this study, we assessed the age‐dependent relationship between cholesterol increase and CRC progression using human CRC samples grouped according to patient age and cancer grade with a machine learning analysis of digitized whole images and retrospective evaluation. Furthermore, we investigated the effectiveness of SQLE and the critical players in the β‐catenin pathway (p53 wild‐type: p53^WT^, p53 mutant: p53^MT^, and the GSK3β activity inhibition, as measured by the phosphorylation at serine 9 of GSK3β: GSK3β^pS9^) as prognostic and diagnostic biomarkers in high‐risk CRC patients.

## 2 RESULTS

### 2.1 Characteristics of the discovery cohort

Table 1 summarizes the clinicopathological characteristics of the discovery cohort. Out of the 1638 specimens, 1311 (80%) were from CRC patients, with a median age of 58 years (range: 24–86 years), consisting of 828 men (63.1%) and 483 women (36.9%). At the time of diagnosis, 646 (49.3%) were in Grade 2, and 162 (12.4%) were in Grade 3. This cohort also included 327 normal colon tissues (20%) to assess the diagnostic potential of the candidates. Additionally, we randomly divided the discovery cohort into training (70%) and testing sets (30%) using a random forest‐based machine‐learning approach (Figure S1).

**TABLE 1:** Clinicopathological characteristics of the discovery cohort.

Discovery phase	Case–control cohort		
All patients	1638		
Cohort size		Patients with cancer	Control subjects
	*n* (%)	1311 (80.0%)	327 (20.0%)
Sex		Patients with cancer	Control subjects
Male	*n* (%)	828 (63.1%)	201 (61.5%)
Female		483 (36.9%)	126 (38.5%)
Age	Overall	Patients with cancer	Control subjects
Mean	57.1	57.4	56.3
Median ± SD^a^	58 ± 13.9	58 ± 13.5	66 ± 15.3
Range (minimum–maximum)	24–86	24–86	25–82
Subtype		Patients with cancer	Control subjects
Adenocarcinoma	*n* (%)	1156 (88.2%)	‐
Mucinous adenocarcinoma		145 (11.1%)	‐
Papillary carcinoma		9 (0.7%)	‐
Signet‐ring cell carcinoma		1 (0.0%)	‐
Control		‐	327
AJCC stage		Patients with cancer	Control subjects
S1–S2	*n* (%)	471 (35.9%)	‐
S3–S4		800 (61.0%)	‐
Unknown		40 (3.1%)	‐
Control		‐	327
The TNM staging		Patients with cancer	Control subjects
T3N0M0 (T1N0M0, T2N0M0)	*n* (%)	213 (16.2%)	‐
T3N1M0 (T3N1M1)		295 (22.5%)	‐
T3N2M0 (T3N2M1)		193 (14.7%)	‐
T4N0M0 (T4N0M1)		218 (16.6%)	‐
T4N1M0 (T4N1M1)		145 (11.1%)	‐
T4N2M0 (T4N2M1)		158 (12.1%)	‐
Unknown		89 (6.8%)	‐
Control		‐	327
Tumor differentiation		Patients with cancer	Control subjects
G1	*n* (%)	396 (30.2%)	‐
G2		646 (49.3%)	‐
G3		162 (12.4%)	‐
Control		107 (8.1%)	‐
Follow up (years)	Overall	Patients with cancer	Control subjects
Mean	7.8	7.6	8.5
Median ± SD	9.2 ± 3.6	9.1 ± 3.6	10.3 ± 3.4
Range (minimum–maximum)	0.2–13.8	0.2–13.8	2.5–13.8

^a^ represents the standard deviation.

### 2.2 The association between cholesterol increase with age and heightened CRC progression

Recently, we demonstrated that lowering SQLE due to cholesterol expedites CRC progression (Jun et al., 2021). This acceleration is facilitated by activating the β‐catenin oncogene and suppressing the p53‐p21 pathways. This study assessed the expression levels of candidates (squalene epoxidase: SQLE, p53 wild type: p53^WT^, p53 mutant: p53^MT^, and GSK3β activity inhibition measured by phosphorylation at serine 9 of GSK3β:GSK3β^pS9^) according to patients' age and CRC aggressiveness.

**FIGURE 1:**
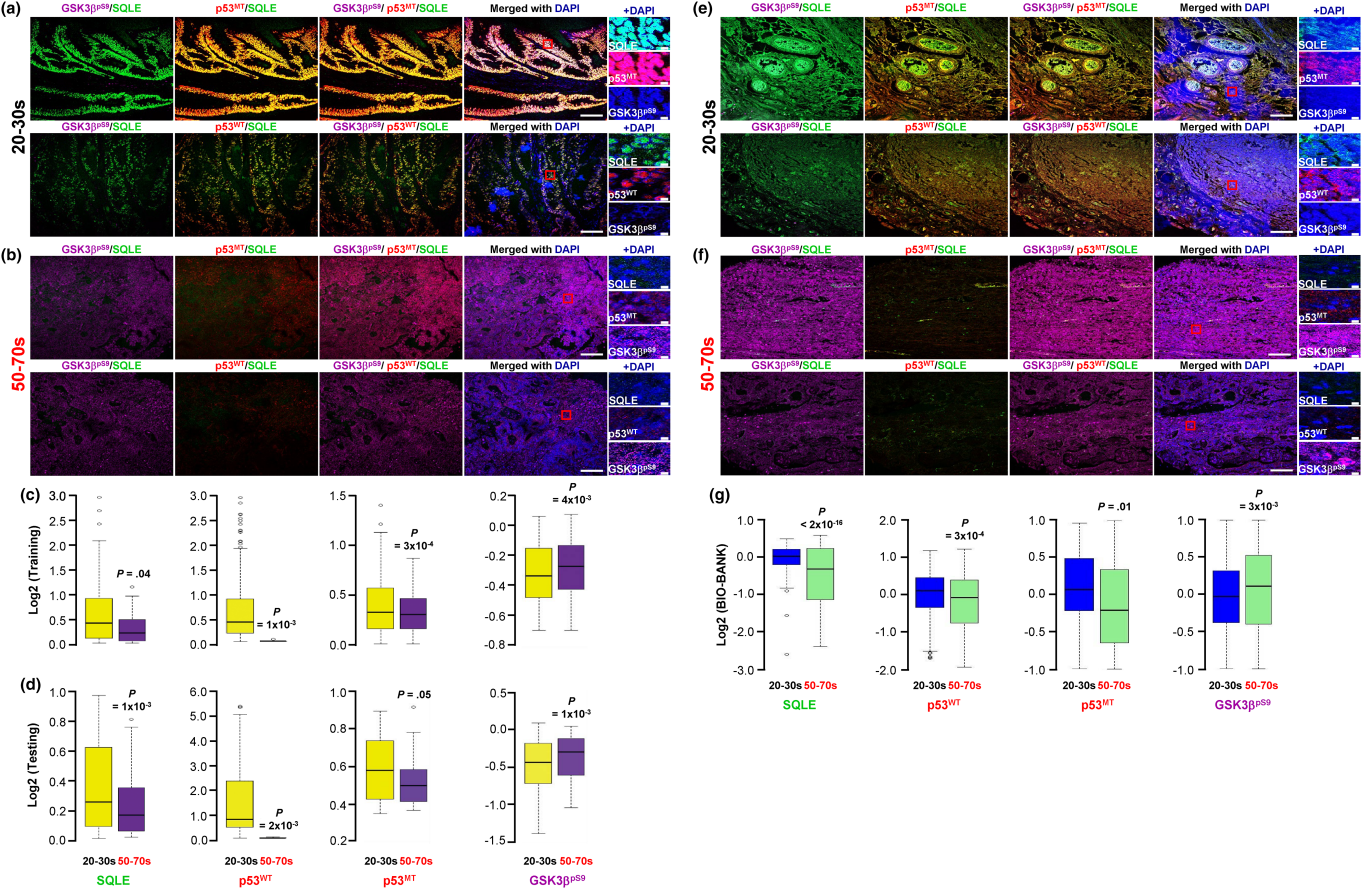
Decreases in SQLE, p53^WT^, and p53^MT^ levels, and an increase in GSK3β^pS9^ in CRCs with aging. CRC specimens obtained from TissueArrary (a–d) or BIO‐BANK (e–g) stratified by patient age were stained with antibodies against the indicated candidate. (a, b, e, f) Each image displays either an overlay of the indicated candidates (100 μM) or a single candidate (5 μM). The red box represents the magnified region (a, b and e, f). Quantification of the candidate levels for multiplex immunohistochemistry (a, b and e, f) was performed (for details, see Materials and Methods; c, d and g). We used p53 (DO‐1) primarily to detect wild‐type p53 (p53^WT^) and p53 (Y5) to determine mutant p53 (p53^MT^). Additionally, the anti‐GSK3β^pS9^ antibody was employed to assess the level of the inactive form of GSK3β. *p values were determined using an unpaired two‐sided *t* test. CRC, colorectal cancer; SQLE, squalene epoxidase.

Multi‐color immunofluorescence staining revealed significant decreases in SQLE, p53^WT^ and p53^MT^, along with an increase in GSK3β^pS9^, in patients aged 50–70 compared to those aged 20–30 (Figure 1a–d). These findings were further validated in a separate BIO‐BANK cohort (Figure 1e–g). Additionally, we confirmed our previous report (Jun et al., 2021), demonstrating the activation of the β‐catenin pathway through SQLE reduction according to CRC progression (Figure S2). Notably, thyroid cancer tissue stained with antibodies also used for CRCs extended our previous findings (Jun et al., 2021), underscoring that SQLE reduction, GSK3β inhibition, and p53 degradation due to aging and increased CRC malignancy are specific events in gastrointestinal cancers (Figure S3).

**FIGURE 2:**
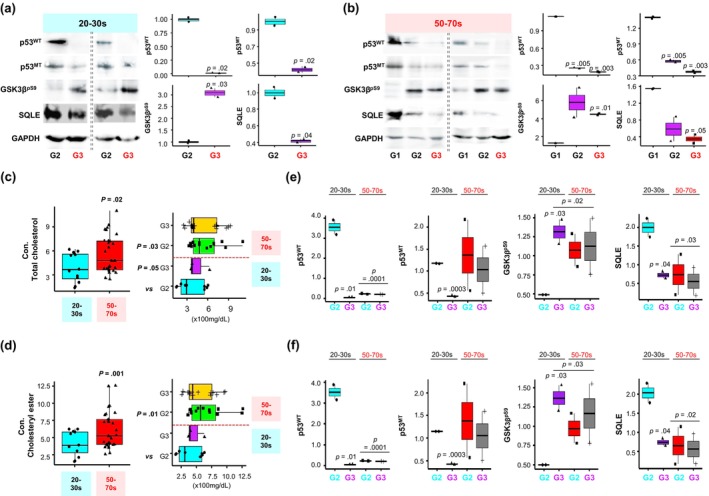
The acceleration of CRC progression via the reduction of SQLE and subsequent activation of the β‐catenin pathway facilitated by aging‐dependent cholesterol accumulation. (a, b) Western blot analysis was performed using ten independent human CRC specimens. GAPDH served as the loading control (left). The expression levels were quantified (right) using ImageJ (NIH, USA). Total cholesterol (c) and cholesteryl ester (d) levels in CRC tissue lysates were measured following the manufacturer's instructions. Each dot in the plot represents a single CRC sample. (e, f) Each candidate's level according to CRC progression was recalculated in relation to the total cholesterol (e) and cholesteryl ester (f) concentration in each age group. *p*‐values were determined by comparing the levels of G2 (a), G1 (b), and G2 20–30s CRC patients (c–f) using an unpaired *t* test. p53^WT^ primarily detects wild‐type p53 (DO‐1), p53^MT^ primarily detects mutant p53 (Y5), and GSK3β^pS9^ represents the inactive form of GSK3β. CRC, colorectal cancer; SQLE, squalene epoxidase.

Our investigation, using tissue lysates categorized by patient age and cancer grade, corroborated a significant reduction in SQLE, p53^WT^, and p53^MT^ and increase in GSK3β^pS9^ levels in malignant CRC tissues (Figure 2a,b). Interestingly, we identified elevated total cholesterol and cholesteryl ester accumulation in aged CRC tissue (Figure 2c,d). Next, to better understand the relationship between cholesterol increase and the activation of the β‐catenin pathway via SQLE reduction, we evaluated the candidate levels in relation to total cholesterol and cholesteryl ester contents normalized by CRC progression and patient age (Figure 2e,f). We did not observe a buildup of total cholesterol and cholesteryl ester in advanced G3 CRC (Figure 2c,d) caused by the degradation of SQLE, which is involved in endogenous cholesterol biosynthesis (Figure 2a,b). Nevertheless, in aged and malignant CRCs, we found significantly reduced SQLE, p53^WT^ and increased GSK3β^PS9^ levels (Figure 2e,f). Notably, p53^MT^ did not exhibit age‐dependent reduction despite its CRC grade‐dependent reduction due to the range of the used antibody, which detecting not only the R273H p53 variant but also others unrelated to the transactivation region of p53 (Figure 2e,f).

Overall, this study establishes a direct association between age‐related tissue cholesterol accumulation and accelerated CRC progression in humans, mediated by the subsequent reduction of SQLE and activation of the β‐catenin oncogenic pathway.

### 2.3 Clinical value of the candidates for a high‐risk CRC population

**FIGURE 3:**
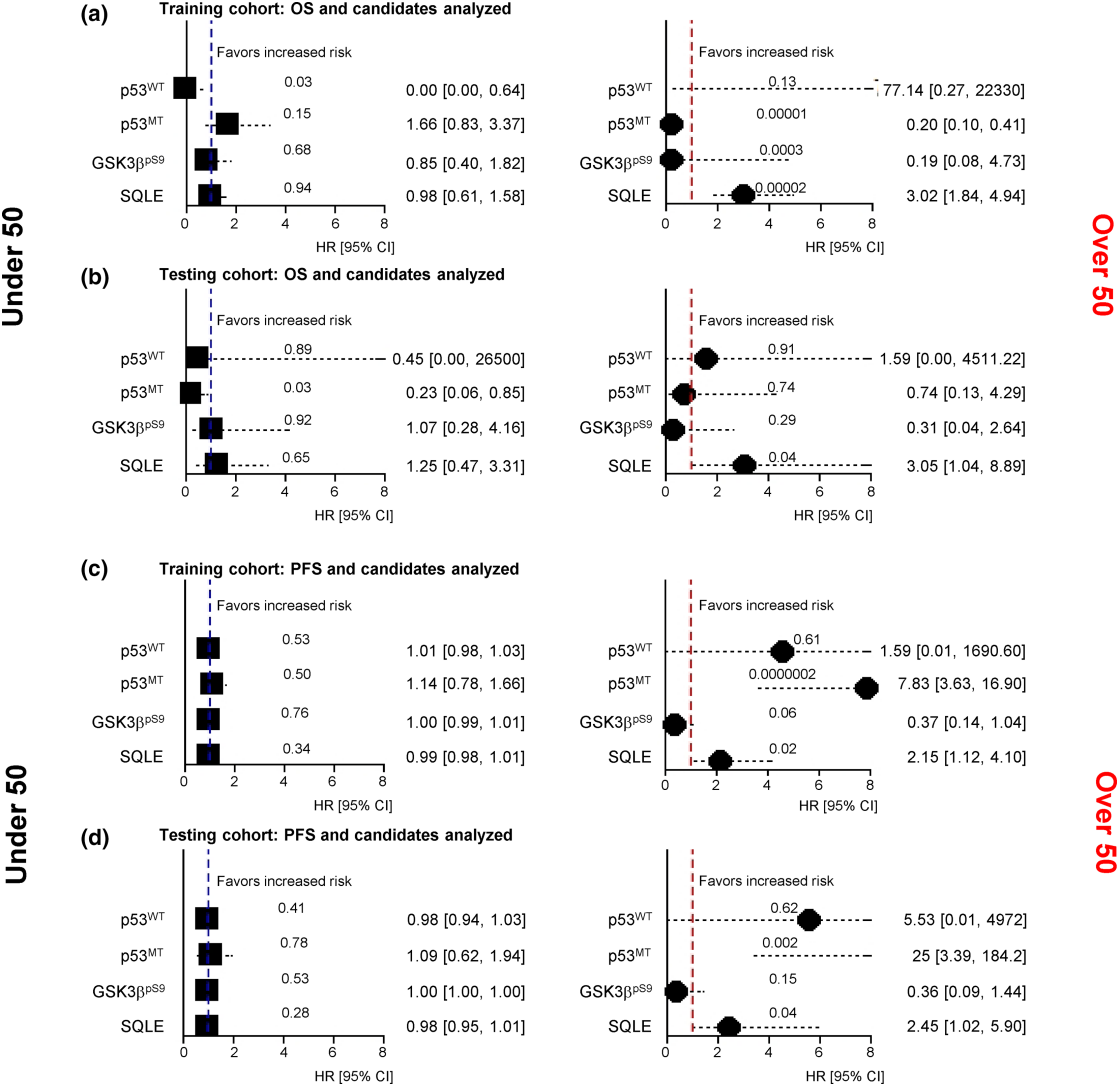
Effects of the candidates with overall survival (OS) and progression‐free survival (PFS) in aged high‐risk colorectal cancer populations. Forest plots for hazard ratios (HRs) based on Cox proportional hazards regression for two groups: before (left) and after (right) 50 years of age, following grades 2 and 3 adjustment. The relationship between OS (a, b), PFS (c, d), and the continuous candidates was analyzed. *p* values are indicated on the dashed line of HR weights. (a, c) Training and (b, d) testing.

Next, we evaluated the candidate's impact on the survival of a high‐risk CRC population, adjusted for grades 2 and 3. Notably, SQLE showed a significant prognostic effect on overall survival (OS) (Figure 3a, right: HR, 3.02, [95% CI, 1.84–4.94], *p* = 0.00002) and progression‐free survival (PFS) (Figure 3c, right: HR, 2.15, [95% CI, 1.12–4.10], *p* = 0.002) on the training set of the aged high‐risk CRC population. These significant findings for SQLE were further validated in the testing set (OS, Figure 3b, right: HR, 3.05 [95% CI, 1.04–8.89], *p* = 0.04; PFS, Figure 3d, right: HR, 2.45 [95% CI, 1.02–5.90], *p* = 0.04). Of note, we discovered a superior effect of p53^MT^, which is SQLE's binding partner, on PFS in the aged population (Figure 3c, right: HR, 7.83 [95% CI, 3.63–16.90], *p* = 0.0000002; Figure 3d, right: HR, 25.00 [95% CI, 3.39–184.20], *p* = 0.002; training and testing set, respectively); however, a 95% CI range of p53^MT^ in the testing set is relatively wide, implying a possible failure to confirm the result in the training set.

**FIGURE 4:**
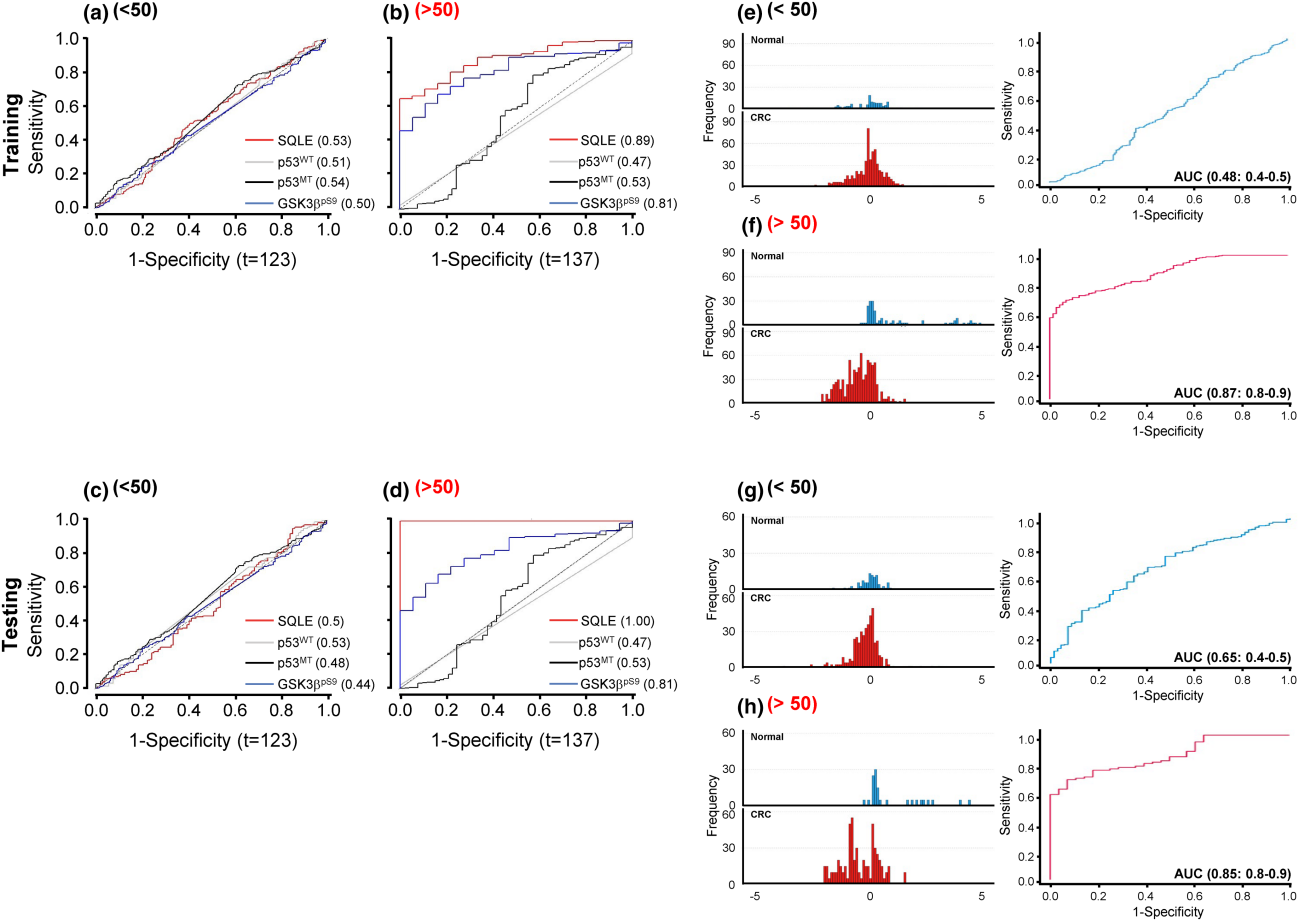
Prognostic and diagnostic performance of the continuous candidates in the high‐risk CRC population. (a–d) For prognostic analysis, time‐dependent ROC analysis was employed, with time intervals determined by maximizing the ROC curve through weighted Cox regression. The time intervals for patients before (left) and after (right) the age of 50 were 123 and 137, respectively. (e–h) For diagnostic analysis, linear discriminant distribution for the merged candidates (left) and ROC curves using the discriminant score for the merged candidate to diagnose CRC (right) are presented. The ratios of AUC, along with their corresponding 95% CIs, are provided. Training sets are depicted in a, b and e, f, while testing sets are shown in c, d and g, h. AUC, area under the ROC curve; CRC, colorectal cancer.

We then evaluated the candidates' ability to distinguish between patients who would survive or die within a given time (*t*). For this purpose, we utilized a time‐dependent ROC (timeROC) analysis, employing a weighted Cox regression for survival data to determine the area under the ROC curve (AUC) (Kamarudin et al., 2017): the times that maximized the AUC ratio were found to be 123 months for patients before the age of 50 and 137 months for those aged 50 and older (Figure 4a–d). Both SQLE (Figure 4b, AUC, 0.89; Figure 4d, AUC, 1.00; training and testing set, respectively) and GSK3β^pS9^ levels (Figure 4b,d, AUC, 0.81; training and testing set) demonstrated outstanding prognostic performance in the population aged 50 years and older. However, this prognostic power was not validated in individuals under 50 (Figure 4a,c).

Additionally, we found that SQLE (AUC, 0.81 [95% CI, 0.8–0.9]) and GSK3β^pS9^ (0.70 [95% CI, 0.7–0.8]) demonstrated sufficient diagnostic efficiency in distinguishing CRCs from controls, in contrast to p53^WT^ and p53^MT^ (Figure S4a,b). These exceptional efficiencies of SQLE (AUC, 0.83 [95% CI, 0.8–0.9]) and GSK3β^PS9^ (AUC, 0.70 [95% CI, 0.7–0.7]) were further confirmed by using ROC curves utilizing the discriminant score (Figure S4c). These findings were validated in the testing set (SQLE, AUC, 0.81 [95% CI, 0.8–0.9]; GSK3β^pS9^, AUC, 0.70 [95% CI, 0.7–0.8]; Figure S4d).

Of note, SQLE (AUC, 0.84, 0.78 [95% CI, 0.8–0.9, 0.7–0.8]; AUC, 0.81, 0.76 [0.7–0.8, 0.6–0.8, before and after age 50, respectively]; training and testing set, respectively) showed excellent CRC diagnosis ability, but it was also confirmed that GSK3β^pS9^ (AUC, 0.66, 0.82 [95% CI, 0.6–0.7, 0.7–0.8]; AUC, 0.67, 0.80 [0.6–0.7, 0.7–0.8, before and after age 50, respectively]; training and testing set, respectively) showed exceptional CRC diagnosis ability in individuals aged over 50 (Figures S5 and S6). Furthermore, the discriminant distribution analysis using the discriminant scores generated from the combined candidates for the older populations exhibited a significant differential distribution (left) and demonstrated excellent diagnostic efficiency (right; AUC, 0.87 and 0.85 [95% CI, 0.8–0.9]; training and testing sets, respectively) (Figure 4f,h), in contrast to the population under the age of 50 years (Figure 4e,g).

## 3 DISCUSSION

This study reveals the correlation between cholesterol accumulation in aging tissue and CRC progression. It also underscores the significant clinical relevance of SQLE, particularly within high‐risk CRC populations, notably among individuals over 50, where there's a distinct increase in both CRC incidence and mortality rates. Furthermore, this study provides valuable biomarkers—SQLE and GSK3β^pS9^, either independently or in conjunction with p53^WT^ and p53^MT^—for risk stratification in populations facing heightened mortality risks due to elevated cholesterol levels.

Our previous proof‐of‐concept demonstration (Jun et al., 2021) showed a cause‐and‐effect relationship between cholesterol increase and CRC progression. However, this relationship was primarily demonstrated in cell lines and animals. Thus, our demonstration needed to be further corroborated in humans.

The decline in CRC incidence, which had been decreasing annually by 3%–4% during the 2000s, slowed to 1% annually between 2011 and 2019 (Siegel et al., 2023). Consistently, this study identified increased CRC incidence and mortality using the Surveillance, Epidemiology, and End Results (SEER) Program's Public database between 2017 and 2019 (Figure S7a,b). This deceleration is primarily attributed to a rise in cases among individuals under 50 years old and in lifestyles characterized by high‐cholesterol diets (Siegel et al., 2023; Xi & Xu, 2021). Our observation, in alignment with others', underlines age‐dependent trends in both CRC incidence and mortality and cholesterol increase (Figures S7c,d and S8). Nevertheless, establishing a cause‐and‐effect relationship between increased cholesterol and CRC incidence and mortality has not been previously determined. This study convincingly demonstrates the SQLE‐mediated link between cholesterol accumulation with age and CRC progression.

Numerous studies have reported the association between obesity and CRC (Mandic et al., 2023; Yang et al., 2023; Ye et al., 2020). SQLE, which is the first oxygen‐requiring enzyme involved in cholesterol biosynthesis, is upregulated during hypoxic conditions in premalignant formation (Coates et al., 2023). Nevertheless, cells tend to favor lipid uptake over internal biosynthesis systems in oxygen and nutrient deprivation during cancer progression. Villa et al. (2016) further supported this notion by demonstrating that glioblastoma cells exhibit increased reliance on external cholesterol by elevating the expression of the low‐density lipoprotein receptor (LDLR) while inhibiting internal cholesterol production. We consistently revealed the elevated expression of LDLR alongside the downregulation of adenosine triphosphate (ATP)‐binding cassette transporter 1 (ABCA1) and SQLE in the progression of gastrointestinal cancers (Jun et al., 2021). This study not only corroborates our previous report but also demonstrated that age‐related accumulation of cholesterol within tissues—potentially through LDLR upregulation and ABCA1 downregulation—accelerates CRC progression via SQLE reduction.

Unlike other genes involved in cholesterol biosynthesis, such as FDFT1, which is upregulated by fasting (Weng et al., 2020), SQLE is reduced when cholesterol accumulates beyond a certain threshold (Gill et al., 2011). Both FDFT1 and SQLE are downregulated during the malignant transformation of CRC. However, while FDFT1 acts as a tumor suppressor by negatively regulating AKT/mTOR/HIF1α signaling in CRC cells (Weng et al., 2020), SQLE activates the β‐catenin oncogenic pathway. Furthermore, the current study demonstrated that SQLE has a clinical impact on high‐risk CRC patients, particularly in the context of cholesterol accumulation associated with aging.

The Wnt/β‐catenin signaling pathway plays a crucial role in CRC development, progression, metastasis, and recurrence. Its abnormal activation is frequently observed in most CRC patients (Chen et al., 2023). Despite this, drugs targeting this pathway have primarily been utilized in the preclinical stage. This study expands upon our previous findings by uncovering the underlying mechanisms linking age‐dependent cholesterol accumulation to CRC progression through SQLE reduction and subsequent activation of the β‐catenin/TCF‐LEF pathway. As a result, this study suggests that quantifying SQLE in CRC tissues using machine learning‐based digital image analysis with fluorescence‐multiplex immunohistochemistry (mlDIA‐fmIHC) can aid doctors in predicting whether patients are at an increased risk of CRC or in need of therapy targeting the β‐catenin‐TCF/LEF complex.

Immunohistochemistry (IHC) has significantly broadened its role in disease diagnosis and outcome prediction, revealing crucial molecules relevant to pathogenesis and potential diagnostic or therapeutic targets, particularly in cancer research. However, conventional IHC, limited to single biomarker detection, and the subjective nature of semiquantitative scoring, susceptible to intra‐ and interobserver variability, pose limitations in clinical and research settings. To address these limitations, we employed multiplex immunohistochemistry capable of detecting multiple molecules in conjunction with CellProfiler (https://cellprofiler.org/), an intuitive image analysis tool utilizing machine learning classifiers: a machine learning‐based digital image with fluorescence‐multiplex immunohistochemistry (mlDIA‐fmIHC). This method has reported remarkable quantitative high‐resolution capabilities in assessing protein expression in tissue (Stirling et al., 2021), surpassing traditional methods like ‘Dukes.’

The mlDIA‐fmIHC analysis elucidated the underlying mechanisms linking intra‐tissue cholesterol accumulation due to aging with the progression of CRC. Additionally, ‘R’ and ‘SPSS’ were employed to evaluate the superior diagnostic and prognostic abilities of candidates including SQLE and GSK3β^pS9^ for CRC patients. Our approach integrated machine‐learning‐based quantitative analysis with multiplex immunohistochemistry, overcoming traditional IHC limitations, which are primarily derived from inter‐ and intra‐observer dependency. Furthermore, this method identified potential candidates for risk stratification and prognosis among CRC patients, thereby ensuring research objectivity and offering valuable avenues for patient care.

Overall, this study effectively resolved the existing controversy surrounding cholesterol and CRC (Fang et al., 2021), confirming that CRC progression accelerates due to cholesterol accumulation within tissues with aging. Furthermore, although large‐scale, long‐term retrospective studies are still required, our research offers valuable biomarkers, including SQLE, for stratifying populations at higher risk of CRC.

## 4 MATERIALS AND METHODS

### 4.1 Sources of patients' samples

This study utilized a total of 1638 formalin‐fixed paraffin‐embedded (FFPE) CRC tissues obtained from TissueArray.com (formerly known as US Biomax, Derwood, MD, USA), constituting the discovery cohort. The characteristics of this cohort, presented in Table 1, were sourced from TissueArray.com. Additionally, we acquired 60 extra FFPE CRC samples and 10 frozen CRC samples from BIO‐BANK (Chungnam National University Hospital, Daejeon, South Korea).

Our inclusion criteria encompassed primary colorectal cancer and normal colon tissues, along with pertinent clinical information about the patients of origin, which were retrieved from the source. The studies using specimens from TissueArray.com and BIO‐BANK received exemptions from the Public Institution Bioethics Committee (designated by the Ministry of Health and Welfare, as well as the Research Ethics Committee of the Korean Research Institute of Bioscience and Biotechnology: exemption codes P01‐202104‐31‐006 and 2021‐0838‐001, respectively). The analyses were conducted between August 2012 and December 2022. This study adhered to the Strengthening the Reporting of Observational Studies in Epidemiology (STROBE) statement (Cuschieri, 2019).

### 4.2 Multiplex fluorescence‐based immunohistochemistry

This study employed multiplex fluorescence immunohistochemistry to evaluate levels of SQLE, p53 wild‐type (p53^WT^), p53 mutant (p53^MT^), and phosphorylation at serine 9 of GSK3β (GSK3β^pS9^) (collectively, “candidates”) within human tissues, following a previously described method (Jun et al., 2021).

In summary, we processed deparaffinized and antigen‐retrieved slides using a series of xylene, ethanol, and citrate‐based buffers. These slides were then incubated overnight with antibodies against SQLE, p53^WT^, GSK3β (Santa Cruz Biotechnology, Dallas, TX, USA; SC‐99144, SC‐126, SC‐377213, respectively), p53^MT^ (Abcam, Boston, MA, USA; ab32049), and GSK3β^pS9^ (Cell Signaling Technology, Danvers, MA, USA; 9323s). Next, each slide was treated with Alexa Fluor‐633 goat anti‐mouse and anti‐rabbit, ‐546 goat anti‐mouse, ‐568 goat anti‐rabbit, or 488 goat anti‐mouse antibody (Thermo Fisher Scientific, Waltham, MA, USA; A21071, A21052, A11030, A11011, or A11001, respectively). After that, we conducted confocal observations using LSM800 (Carl Zeiss, Oberkochen, Germany). All procedures were performed by qualified personnel at the Korea Research Institute of Bioscience and Biotechnology pathology tissue core facility.

To affirm antibody specificity, we also stained thyroid cancer tissues with the same antibodies used for CRCs (Figure S1). This staining confirmed the age‐dependent reduction of SQLE and subsequent the β‐catenin pathway activation is a characteristic feature in gastrointestinal cancers (Jun et al., 2021; “The Human Protein Atlas: https://www.proteinatlas.org/ENSG00000188404‐SELL/pathology”).

### 4.3 Analyzing and quantifying images

The process of digital image analysis using CellProfiler's 4.2.6 pipeline involved the following steps:

The staining results for each candidate antibody on a tissue slide were labeled using specific names based on the name and type mode. Next, all fluorescence images requiring quantification were converted to grayscale using the ColorToGray mode. Then, in the threshold mode, each image underwent adjustments using the Global threshold strategy, incorporating the Otsu and two‐class threshold methods (with settings including a threshold smooth scale of 0.2, a threshold correction factor of 2.0, and thresholds ranging from 0.2 to 2.0). After the ImageMath phase, advanced settings were employed in the IdentifyPrimaryObjects stage. These included setting the typical object diameter in pixel units from 1 to 2000, allowing objects outside this range and those touching the image border to be discarded. The method chosen to distinguish and draw dividing lines between the clumped objects was “Intensity.” Additionally, automatic calculation of the minimum allowed distance between local maxima was disabled, the size of the smooth filter was set to 10, local maxima closer than the minimum distance of 5 were suppressed, and lower‐resolution images were used to expedite finding the local maxima without displaying the accepted local maxima. Identified object gaps were filled ‘After both thresholding and declumping,’ and if an excessive number of objects were detected, the system was set to ‘Continue.’ Finally, in the MeasureObjectIntensity phase, quantification of each staining on a tissue slide was executed based on specific conditions involving the selection of images and objects for measurement.

For the comparative analysis using the SQLE, p53^WT^, p53^MT^, and GSK3β^pS9^ values obtained through the CellProfiler, a global normalization approach was applied as follows: each candidate's value was divided by the median intensity of GSK3β, followed by log transformation (Idikio, 2011). Subsequently, the individual log value of each candidate was subtracted from the median log value. The resulting candidate levels are presented as the median with the standard error of the mean. Statistical significance for all measurements was assessed through an unpaired two‐sided *t* test utilizing R software.

### 4.4 Measurement of intra‐tissue total cholesterol and cholesteryl ester content

The intra‐tissue total cholesterol and cholesteryl ester contents were determined according to the manufacturer's instructions (Cholesterol/Cholesteryl Ester Quantitation Assay kit: abcam, ab65359). Briefly, CRC tissues were homogenized in a mixture (chloroform: isopropanol: NP‐40; 7:11:0.1, respectively). After centrifugation at 15,000*g*, the liquid phase was vacuum air‐dried at 50°C for 30 min. The reaction mixture with and without cholesterol esterase was mixed with the extracted sample to measure the total cholesterol and free cholesterol in the assay buffer. Total and free cholesterol levels were determined by incubating the reaction mixture at 37°C for 60 min and measuring the absorbance at the OD570nm. Cholesteryl ester concentration was calculated by subtracting the free cholesterol value from the total cholesterol value.

To evaluate the candidate levels in CRC progression with patient age, each value for the candidate staining was divided by the value of GAPDH. For normalization between ages, each value was then divided by the highest number among 20–30s and 50–70s. After that, each value was multiplied by the level of total cholesterol and cholesteryl ester in each grade and age group.

### 4.5 Western blotting

Western blotting was conducted as previously described (Jun et al., 2021). To summarize, tissue lysates were prepared in ice‐cold Pierce IP Lysis/Wash Buffer (Thermo Fisher Scientific, 87788). The lysates were subsequently separated by sodium dodecyl sulfate‐polyacrylamide gel electrophoresis and transferred onto nitrocellulose membranes. The membranes were then incubated with the indicated antibodies, which were also used for multiplex fluorescence‐based immunohistochemistry. The specificity of these antibodies was evaluated in our previous report (Jun et al., 2021).

### 4.6 Cox analysis

Multivariable Cox proportional hazard analysis was conducted to assess the impact of the candidate on the OS (Figure 3a,b) and PFS (Figure 3c,d) of grade 2–3 patients, stratified by age groups: those aged 50 and below (left) and those aged above 50 (right). The analyses were conducted using the survival package in R. *p* values are noted in the dashed lines.

### 4.7 Prognostic and diagnostic analysis

To assess the candidates' prognostic and diagnostic abilities, we obtained information on OS and PFS for cancer and non‐cancer patients from the supplier (Tissuearray.com) between August 2012 and June 2020. The analyses concluded in December 2022.

For the prognostic analysis, we performed a time‐dependent receiver operating characteristic (timeROC) analysis using a weighted Cox regression for survival data to determine the area under the ROC curve (Figure 4; Kamarudin et al., 2017). We also used ROC curves with or without a discriminant score for the diagnostic analysis. We performed timeROC analysis and ROC curves without a discriminant score using R, whereas ROC curves with a discriminant score were using SPSS. Survival analyses were conducted using the multivariate Cox proportional hazards methods (Figure 3). Survival analyses were performed using the multivariate Cox proportional hazards approach in R. Statistical significance for all measurements was assessed through an unpaired two‐sided *t* test utilizing R software.

## AUTHOR CONTRIBUTIONS

Jun SY made the primary contributions to the study, including conceptualization, conducting experiments, data analysis, data curation, and manuscript writing. Yoon HR assisted in establishing digital image analysis, contributed to data curation, and participated in manuscript writing. Yoon JY and LJJ supported western blot analysis. Kim JY and Kim JM played critical roles in collecting human tissues and analyzing patient information. Kim NS contributed to study conceptualization, data analysis, manuscript writing, *funding acquisition*, project administration, and supervision.

## FUNDING INFORMATION

This work was supported by a Basic Science Research Program grant through the National Research Foundation (NRF) of Korea funded by the Ministry of Science, ICT and Future planning (2020R1A2C2006752) and the KRIBB Research Initiative Program (KGM5222423) in the Republic of Korea.

## CONFLICT OF INTEREST STATEMENT

The authors declare that they have no competing interests.

## Supporting information


Data S1:

## Data Availability

Our imaging metadata includes patient personnel information, such as patient age, birth date, CRC grade, overall survival, progression‐free survival, and so forth. Therefore, we cannot make these data available on public websites. However, we (nskim37@kribb.re.kr) are happy to provide the information upon request and after completing the necessary IRB procedures. We are also delighted to offer analytic methods, such as Cox regression analysis using R (nskim37@kribb.re.kr or gut2040@gmail.com).
