# Supplementary material for: Age‐related cholesterol and colorectal cancer progression: Validating squalene epoxidase for high‐risk cases
Source: Aging Cell. 2024 Mar 22;23(7):e14152. doi: 10.1111/acel.14152 (PMC11258466; doi:10.1111/acel.14152)
Supplement: Supplementary file 1 — Data S1: [file ACEL-23-e14152-s001.docx]

**Age-related cholesterol and colorectal cancer progression: Validating**

**squalene epoxidase for high-risk cases**

by **Soo Young Jun;** Hyang Ran Yoon; Ji-Yong Yoon; Jeong-Ju Lee;

Ji Yeon Kim; Jin-Man Kim; Nam-Soon Kim*

Supplementary figures

8 figures

**
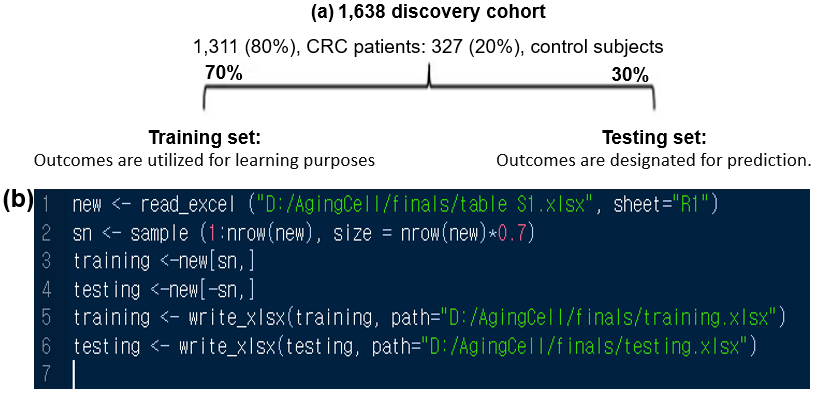
**

**Fig S1. Schematic Representation of Study Design**

**(a)** The 1638 patients from the discovery cohort were divided into 70% training and 30% testing sets using **(b)** the random forest algorithm. **(a)** The training and testing sets were set for learning and prediction purposes, respectively.

**
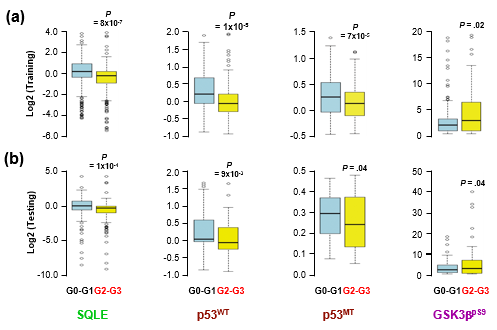
**

**Fig S2. Alteration in SQLE, p53^WT^, p53^MT^, and GSK3β^pS9^ levels according to CRC progression**

CRC specimens, provided by TissueArray, were re-arranged according to CRC grade, stained concurrently with antibodies against the indicated candidate, and observed under confocal microscopy. (a-b) The quantification of each candidate in relation to CRC progression is shown. p53^WT^: wild-type p53 (DO-1), p53^MT^: mutant p53 (Y5), and GSK3β^pS9^: the inactive form of GSK3β determined by using the anti-GSK3β^pS9^ antibody. **P* values were determined using an unpaired *t*-test.


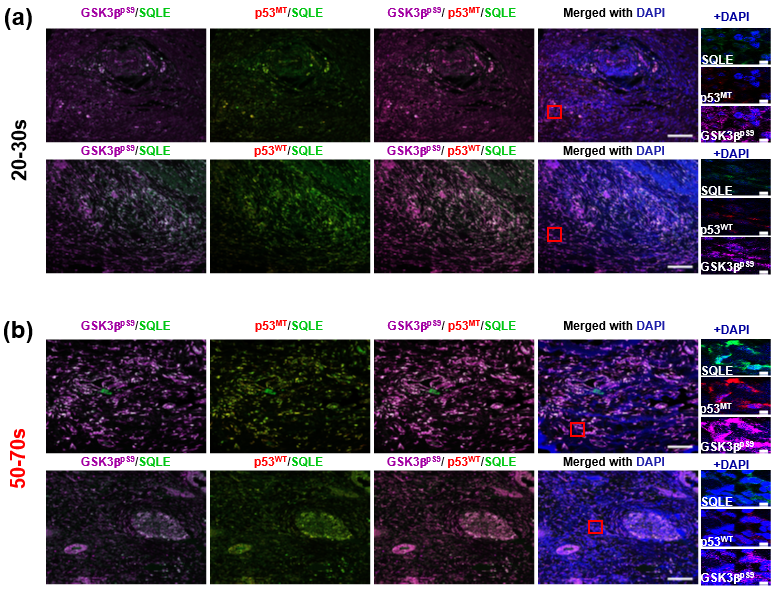


**Fig S3. Expression levels of the candidates in thyroid cancers**

Thyroid cancer tissues, provided by BIO-BANK, grouped before (a) and after (b) the age of 50 years, were stained with antibodies against the candidates and then observed under confocal microscopy. Each image is presented as an overlay of the indicated candidates (100μM) or a single (5μM). The red box shows the regions magnified. p53^WT^; wild-type p53 (DO-1), p53^MT^; mutant p53 (Y5), GSK3β^pS9^: the inactive form of GSK3β (the anti-GSK3β^pS9^ antibody). DAPI was used for nucleus staining.


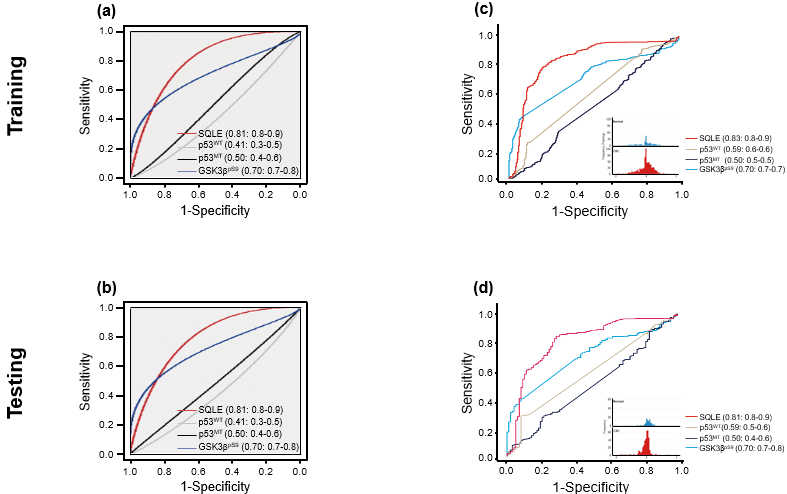


**Fig S4.** **ROC analysis for diagnosing CRCs.**

(a-b) ROC curves for each candidate are shown. (c-d) A linear discriminant distribution for SQLE (insert) and the ROC curves using the discriminant score for individual candidates to diagnose CRC are presented. The ratio of AUC and the corresponding 95% CI are noted.

**
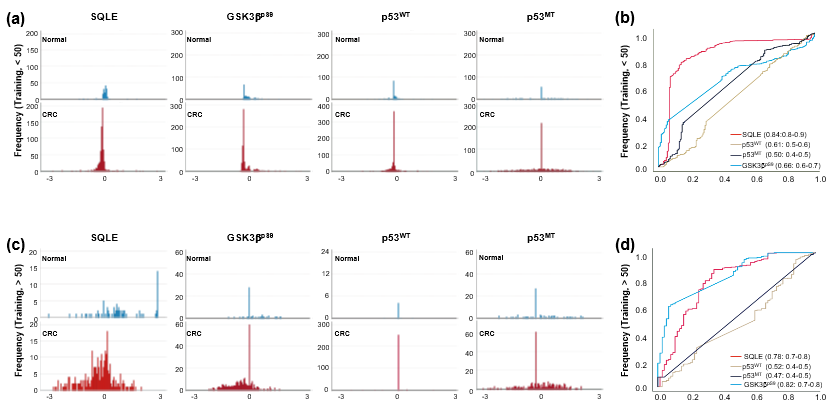
**

**Fig S5. Discriminant distribution and diagnosing potential of the candidate between CRC and normal (Training set)**

(a, c) Linear discriminant distribution analysis for normal (upper) and CRC (lower) patients and (b, d) corresponding ROC curves based on discriminant scores of SQLE, GSK3β^pS9^, p53^WT^, and p53^MT^ in a population divided before (a-b) and after (c-d) the age of 50.

**
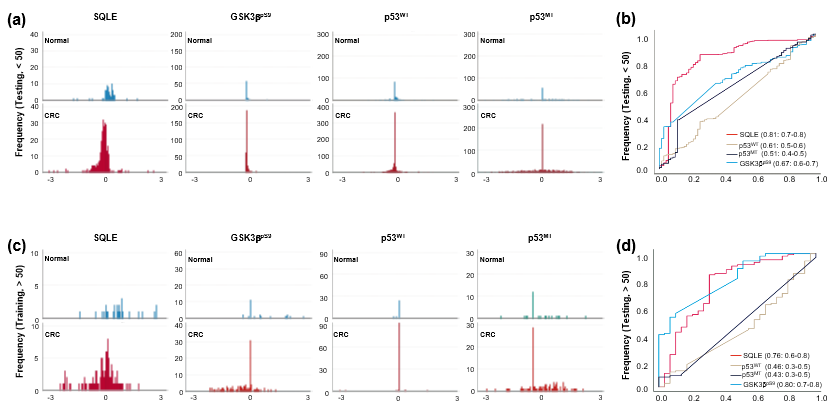
**

**Fig S6. The discriminant distribution and diagnostic potential of the candidate between CRC and normal tissues (Testing set)**

(a, c) Linear discriminant distribution analysis for normal (upper) and CRC (lower) along with (b, d) generating ROC curves utilizing discriminant scores of SQLE, GSK3β^pS9^, p53^WT^, and p53^MT^ within a population divided into age groups: before (a-b) and after (c-d) 50 years of age.

**
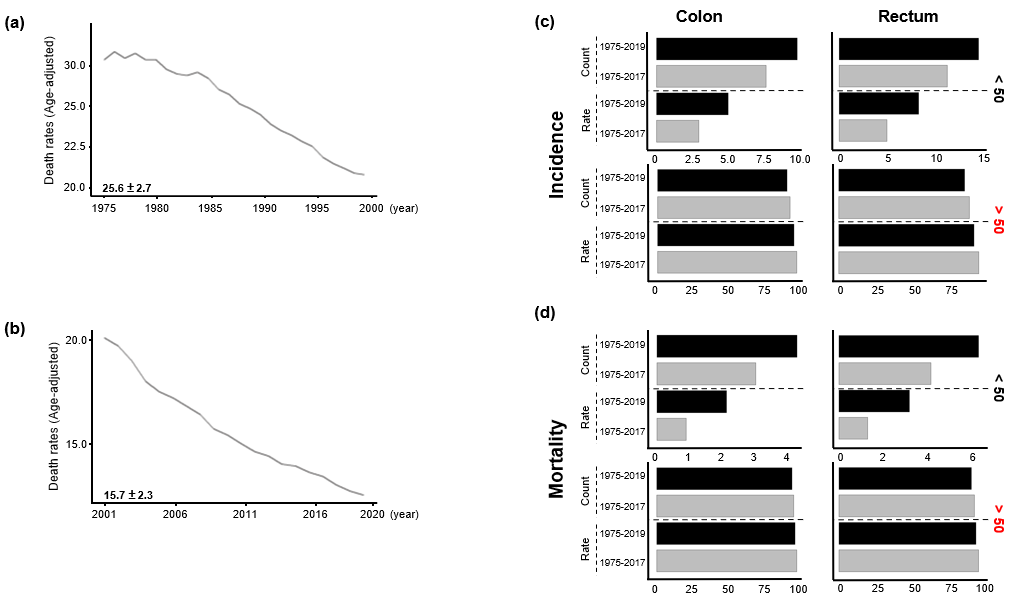
Fig S7. CRC incidence and mortality.** We used data from the SEER cancer statistics review for 1975-2000 (a) and Cancer Stat Facts: Colorectal Cancer (seer.cancer.gov/statfacts/html/colorect. html) for 2001-2020 (b). The numbers (lower left) indicate the median mortality of CRC patients (a, b). CRC Incidence (c) and mortality (d) between patients before and after age 50 (SEER 9 Registries). Incidence rate = (New cancers / Population) x 100,000; Mortality rate = (Cancer deaths / Population) x 100,000. The 2000 US standard population was used for the age adjustment of rates. Incidence rates are adjusted for reporting delays. Source: Incidence – SEER program, 2022. Mortality – NCHS, 2022.

**
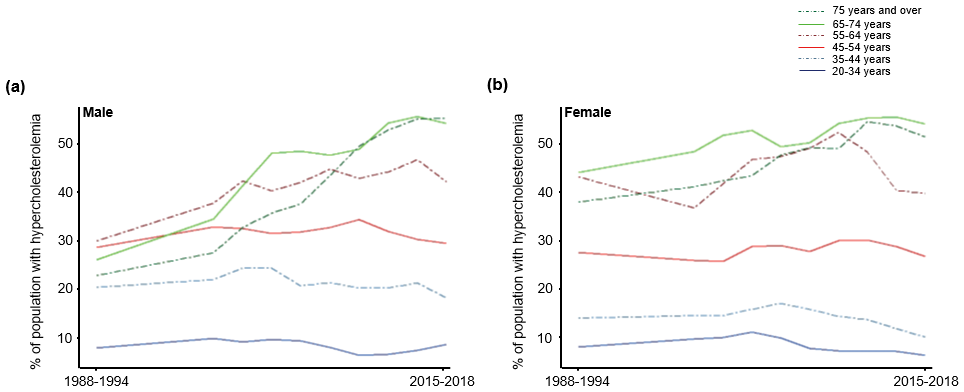
**

**Fig S8. An increasing population percentage with hypercholesterolemia according to aging**

The following years were unmarked: 1999-2002, 2003-2006, 2007-2010, and 2011-2014.

**Notes:** Hypercholesterolemia, also referred to as high total cholesterol, is defined as 240 mg/dL or more. All estimates were age-adjusted using the direct method to the projected 2000 US Census population, with age groups of 20-39, 40-59, and 60 and over. Source: NCHS, National Health and Nutrition Examination Survey, 2015-2018.
